# Silent Disco Headphone Parties in Canadian Long-Term Care Homes

**DOI:** 10.1093/geroni/igaf122.709

**Published:** 2025-12-31

**Authors:** Lillian Hung

**Affiliations:** University of British Columbia, Vancouver, British Columbia, Canada

## Abstract

Silent disco headphone parties offer an innovative way to foster social connection and engagement among older adults in long-term care (LTC) by allowing residents to dance and enjoy music through wireless, multichannel headphones. Unlike traditional group activities, silent discos provide residents with the autonomy to select their preferred music while participating in a shared, joyful and immersive experience. Given the high prevalence of loneliness and social isolation among LTC residents, finding creative approaches to encourage social interaction and meaningful engagement is essential. This study examined the experiences of 180 participants—including residents, family members, and interdisciplinary staff—across three Canadian LTC homes. Data were collected using video ethnography, incorporating video recordings, conversational interviews, direct observations, and focus groups to capture diverse perspectives on the experience. Thematic analysis revealed three central themes: bridging generations, fostering social interaction, and promoting a sense of community. Participants expressed that silent disco parties created opportunities for spontaneous interactions, strengthened connections between residents and caregivers, and facilitated intergenerational engagement. Findings suggest that silent disco parties have the potential to enhance social well-being in LTC by providing an inclusive, accessible, and adaptable social activity that fosters participation and enjoyment. These events may also contribute to reducing feelings of isolation by promoting movement, shared experiences, and emotional expression through music. Future research should further investigate the long-term benefits of silent disco parties on residents’ social health, emotional well-being, and overall quality of life.

